# Digital Twin for a Collaborative Painting Robot

**DOI:** 10.3390/s23010017

**Published:** 2022-12-20

**Authors:** Ratchatin Chancharoen, Kantawatchr Chaiprabha, Lunchakorn Wuttisittikulkij, Widhyakorn Asdornwised, Muhammad Saadi, Gridsada Phanomchoeng

**Affiliations:** 1 Department of Mechanical Engineering, Faculty of Engineering, Chulalongkorn University, Bangkok 10330, Thailand; 2 Department of Electrical Engineering, Faculty of Engineering, Chulalongkorn University, Bangkok 10330, Thailand; 3 Department of Electrical Engineering, Faculty of Engineering, University of Central Punjab, Lahore 54000, Pakistan; 4 Smart Mobility Research Unit, Faculty of Engineering, Chulalongkorn University, Bangkok 10330, Thailand

**Keywords:** digital twin, painting, collaborative robots, Industry 4.0, automation, serial mechanisms, spray gun

## Abstract

A collaborative painting robot that can be used as an alternative to workers has been developed using a digital twin framework and its performance was demonstrated experimentally. The digital twin of the automatic painting robot simulates the entire process and estimates the paint result before the real execution. An operator can view the simulated process and result with an option to either confirm or cancel the task. If the task is accepted, the digital twin generates all the parameters, including the end effector trajectory of the robot, the material flow to the collaborative robot, and a spray mechanism. This ability means that the painting process can be practiced in a virtual environment to decrease set costs, waste, and time, all of which are highly demanded in single-item production. In this study, the screen was fixtureless and, thus, a camera was used to capture it in a physical environment, which was further analyzed to determine its pose. The digital twin then builds the screen in real-time in a virtual environment. The communication between the physical and digital twins is bidirectional in this scenario. An operator can design a painting pattern, such as a basic shape and/or letter, along with its size and paint location, in the resulting procedure. The digital twin then generates the simulation and expected painting result using the physical twin’s screen pose. The painting results show that the root mean square error (RMSE) of the painting is less than 1.5 mm and the standard deviation of RMSE is less than 0.85 mm. Additionally, the initial benefits of the technique include lower setup costs, waste, and time, as well as an easy-to-use operating procedure. More benefits are expected from the digital twin framework, such as the ability of the digital twin to (1) find a solution when a fault arises, (2) refine the control or optimize the operation, and (3) plan using historic data.

## 1. Introduction

The development of digital technologies has reshaped the world. New technologies and methods are being introduced which have revolutionized the way we live today. Not only humans, but industries, factories, and manufacturing processes have evolved through the technological revolution. The industrial revolution has evolved from the mechanical production facilities powered by water and steam to the current cyber-physical production systems, called the Industrial Revolution 4.0 (IR 4.0) [1,2,3]. Industry 4.0 uses automation technologies, artificial intelligence (AI), machine learning (ML), cloud computing, edge computing, fifth-generation (5G) cellular networks, Internet of Things (IoT), big data, etc. to promote potential in every aspect of the industry [4]. Moreover, the use of these automation and digital technologies enables the integration of the physical and digital worlds to create virtual products and processes, resulting in optimized factories and manufacturing processes [5].

The concept of a digital twin is essentially a computer program that uses real-world data to produce simulations to predict the performance and behavior of a system. This concept was first introduced by NASA’s Apollo space program [6], which was then used in a three-dimensional (3D) model and digital concept to design urban road networks [7] and more [8,9,10]. Emulating a real-world system in a virtual world requires both worlds to be synchronized and that can be achieved with the help of sensing devices, connected smart devices, IoT, AI, ML, and real-time data elaboration. The advantage of the digital twin for Industry 4.0 manufacturing systems is to exploit its features to forecast and optimize the behavior of real systems at each life cycle phase in real time [11,12,13,14].

**Figure 1:**
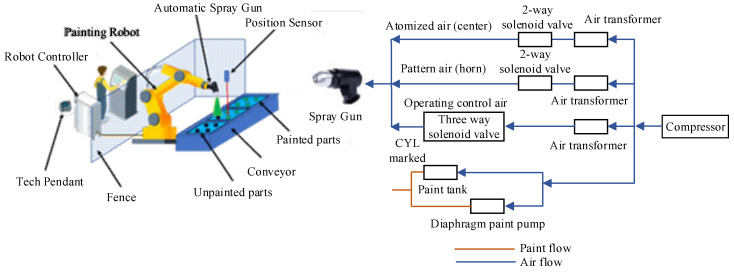
Traditional Painting Robot.

Collaborative painting robots are gaining much attention lately concerning the framework of digital twins and that is in line with the standards and practices of IR 4.0. This is primarily because of improved safety, reduced waste material, superior efficiency and improved system uptime, to name a few, when compared with traditional painting robots [15,16,17]. With rapidly growing economies, industrialization and population growth, new construction is taking place everywhere. Metropolitan cities are developing vertically, and parts of the buildings are sometimes hard to reach. It is therefore a need of the hour to devise new tools which can perform laborious painting tasks efficiently. The traditional painting robot’s system comprises position sensors, an automatic spray gun, and a painting robot as shown in Figure 1 [18,19]. The issues that are encountered by traditional painting robots are unstable trajectory and movement speed that results in bubbles and scars to the painted surface causing poor and uneven-thickness coatings. Furthermore, in a practical environment, it is very hard to optimize massive parameters in a complex system operating in an unknown environment using traditional methodology.

To control the quality and standard of painting, tool planning algorithms and painting models have been developed and researched for many years [20,21,22,23,24]. However, in practice, it is very difficult to generate an online tool trajectory, and obtain the optimal tool trajectory and film quantity deviation for a free-form surface. Automated tool planning is the bottleneck for the painting process. Control strategies for eliminating the dynamic and friction influences and improving the accuracy and repeatability of robots have been developed [15,25,26,27,28,29,30]. However, owing to the complexity and time-intensiveness of the painting process, their developed algorithms seem unable to solve the problem online and cannot solve the trajectory optimization problem.

To overcome the shortcoming and challenges of traditional painting robots, a collaborative painting robot is introduced in this paper. Collaborative robots bring the strength of humans and robots in one place that helps overcome their weaknesses. It offers assistance to human counterparts in performing tedious, dull and dirty jobs. The main contributions of this paper are

- Development and experimental performance evaluation of a collaborative painting robot using a digital twin framework.
- The digital twin of the automatic painting robot simulates the entire process and estimates the paint result before the real execution. This results in decreased set costs, waste, and time, with improved results.

The rest of this article is organized as follows. In Section 2, we present the setup and communication architecture between the physical collaborative painting robot and the virtual system that represents the robot. In addition, a case study task of painting is presented, and the digital twin-based methodology demonstrates how to solve and perform the task. Next, the results and discussion of the proposed approach and its benefits are presented in Section 3 and Section 4, respectively. Finally, the conclusions are presented in Section 5.

## 2. Materials and Methods

### 2.1. Digital Twin Architecture for Collaborative Painting Robot

Painting robots have been used in various applications of the painting industry, such as car and boat painting. Examples of well-known painting robots are the Dürr spray-painting robot EcoRP E043i, ABB spray-painting robot IRB 52, and Fanuc spray-painting robot P-250iB, which are serial mechanisms or 6 degrees-of-freedom (6-DOF) robots [31,32,33,34,35]. Such robots have the advantages of a large workspace, high dexterity and maneuverability, complex movement and pose, speed, and accuracy. Although there are some disadvantages, such as low payload-to-weight ratio, low stiffness, and safety due to their large workspace, they are not issues for painting applications because of the weight of their sprayers. Thus, such robots are well-known for their painting applications.

Most well-known commercial robots may be programmed using three techniques: first, by operators teaching their position and movement; second, by a teaching pendant; and third, by a script run on an external computer. For the traditional programming methods, most operators use the first two techniques to program a robot because they are simple, and operators can confirm the programming with robot simulation on the teaching pendant or the physical movement of the robot. However, with these techniques, the program cannot be quickly changed or modified online. Operators must go to the robot and apply the first or second programming technique and confirm the program with offline simulation or physical movement. The third technique seems more complex for operators because it requires an external computer, complex programming skills, and the program can still not be confirmed with online simulation.

**Figure 2:**
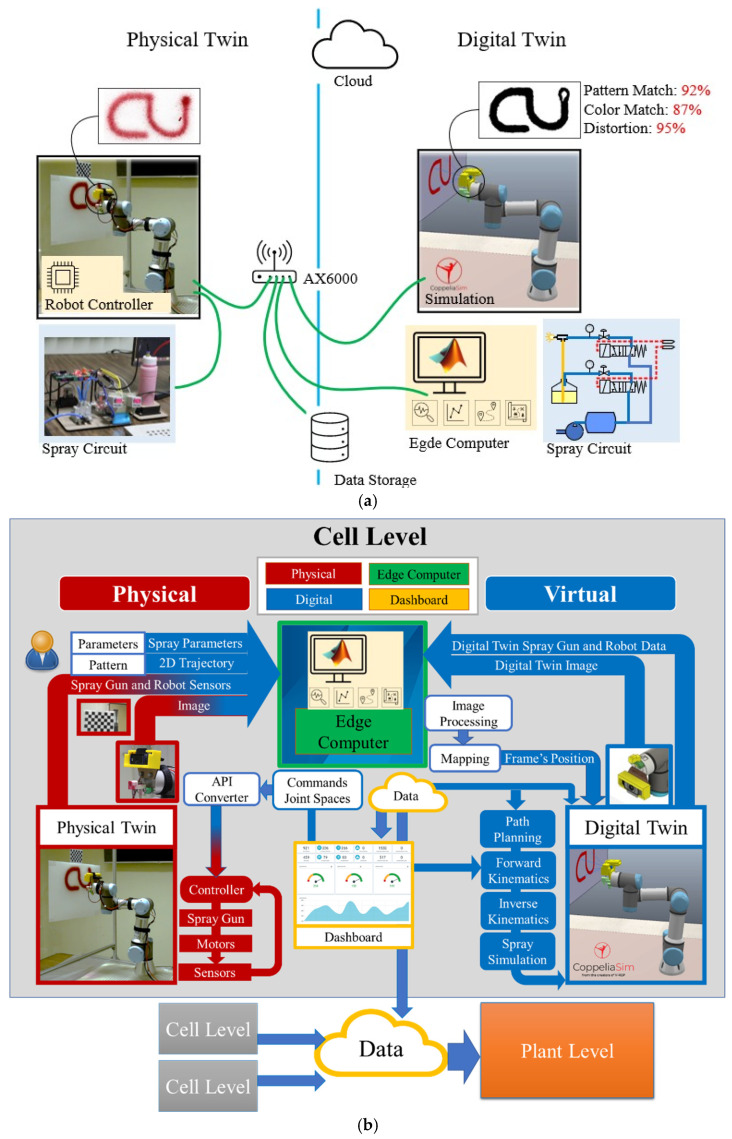
Collaborative Painting Robot with Digital Twin Framework: (**a**) The Proposed Digital Twin Framework and (**b**) Digital Twin Data Flow.

The proposed digital twin framework of the collaborative painting robot with the digital twin is shown in Figure 2. The proposed digital twin architecture and communication model is an open architecture that supports available tools, hardware, and software to build a digital twin for a collaborative robotic cell. The Wifi-AX6000 connects all of the hardware in the automation cell to realize data bandwidth, latency, and massive communication. This supports Wi-Fi IEEE 802.11ax, IEEE 802.3/3u/3ab, and GigE standards, making it suitable for machine communication. CoppeliaSim, a physics simulation and visualization mechanism, runs on a dedicated computer, whereas MATLAB runs on a shared edge computer. The robot’s controller, data storage, and internet are also connected to the network. In this architecture, the physics simulation and edge computer update at different rates. It should be noted that the internet and cloud will be used in the future to connect the cell to higher levels of automation and commercially available cloud services.

The data flow of this digital twin framework is shown in Figure 2b. The edge computer is used for all high-level computing such as design pattern and design trajectory. The low level is the position and velocity control of the robot joints that are controlled by the robot controller. For the virtual representation, the physics simulation is used to simulate the characteristics and motion of a real collaborative painting robot [36,37,38,39]. The characteristic and motion outputs of a robot can be obtained and sent to the edge computer program [40]—edge computer—to process. Moreover, the physics simulation obtains the inputs from the edge computer program and sensor camera to perform the simulation with the current information. The edge computer program is also included in the virtual representation. It works as the controller/edge computer of the system. It obtains the data from the physics simulation, the real robot, and the sensor camera to compute the control action and sends the command to the physics simulation, the real robot, and the spray gun. Then, the real collaborative painting robot, spray gun, and sensor camera are presented as the real system. The sensor camera is used to measure environmental data and send the data to the edge computer program and the physics simulation. The spray gun is used to paint when the command is given. Next, the real collaborative painting robot obtains the command from the edge computer program—controller—and executes the task and feeds back the outputs to the edge computer program. With this architecture, the real-time simulation and control of the collaborative painting robot can be achieved, and the online automated tool planning can be developed in real-time with real-time simulation technology and is accurate. The operators can program the robot easily by designing a desired pattern on the PC/screen. Then, the physics simulation program simulates the process and estimates the results. Once the results are confirmed, the edge computing program creates the projection model and plans for the robot trajectory. Then, the edge computing program sends the designed trajectory to the real robot and commands control of the process flow of the spray mechanism. Next, the robot executes the task. During the painting, the camera is used to monitor, compare the digital twin image and real image, and analyze the error, and sends the feedback to the edge computer. The error can be used to stop the robot or correct the robot’s trajectory in real-time.

**Figure 3:**
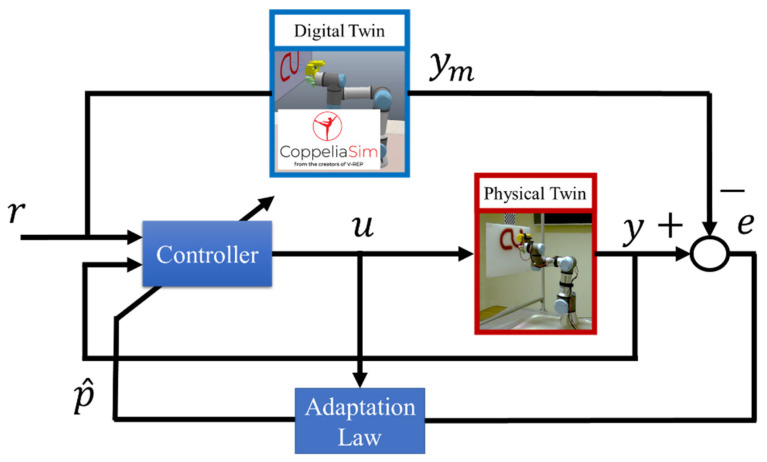
Model Reference Adaptive Control Concept.

This concept can be extended to be the model reference adaptive control concept (Figure 3). The reference input, r, is the desired painting pattern. The outputs, $y_{m}$ and y, are the estimated desired painting pattern output and the actual desired painting output, respectively. Next, the error difference between the estimated and desired painting pattern outputs is defined by e. In addition, the controller command is defined by u and the parameter setting of the system is defined by $\hat{p}$.

### 2.2. Collaborative Painting Robot System Hardware and Components

#### 2.2.1. Collaborative Painting Robot

**Figure 4:**
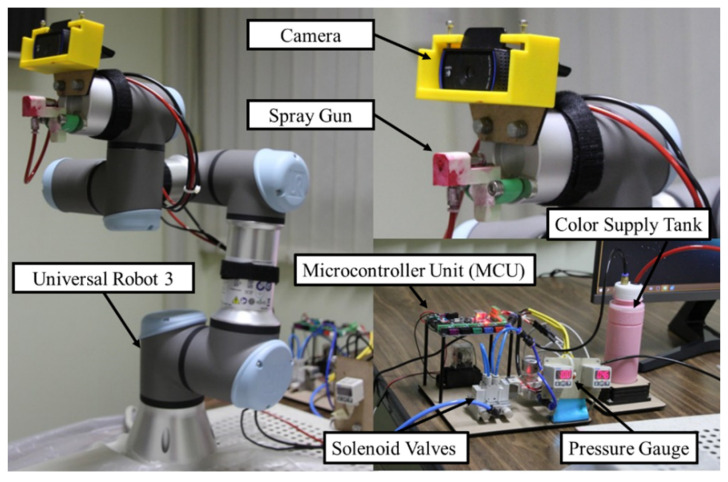
The Components of the Collaborative Painting Robot.

For the collaborative painting robot, a collaborative robot Universal Robot UR3 model was selected to be implemented due to its benefits [2,3,41]. For example, various robot sizes can be selected; thus, they can be applied for various painting applications. In addition, Universal Robots can solve the robot safety issue because they are designed to work with humans in the same space. It is possible to develop an advanced painting application with which humans and Universal Robots can help each other to work in the same space. Moreover, Universal Robots’ ecology is thriving thanks to the popularity of robots among researchers. There are many publications, mathematic models, simulations, and advanced control techniques based on these robots [42,43,44]. The advanced implementation can be developed based on the existing technology. Therefore, this benefit facilitates the development of a digital twin architecture and is essential for developing a highly complex but comprehensive digital twin system. Figure 4 shows the components of the collaborative painting robot.

**Figure 5:**
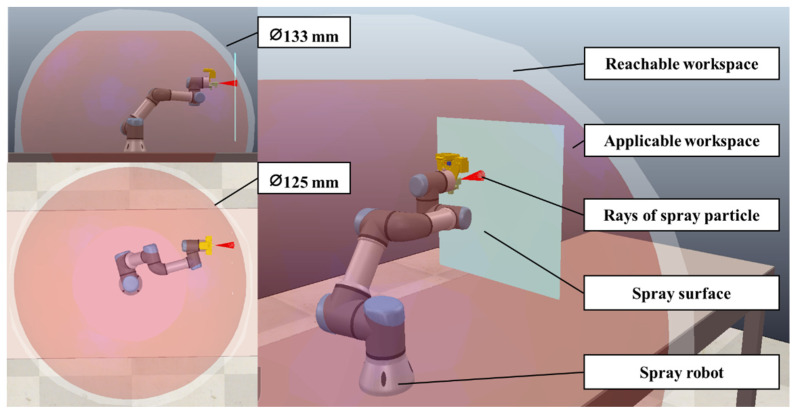
The Workspace of the Robot for the Painting Application.

The collaborative robot, a 6-DOF manipulator robot, belongs to the UR family of Universal Robots [45]. The controller software of the collaborative robot is Universal Robots 3.15. The workspace of the collaborative robot for the painting application is shown in Figure 5. The red area is the applicable workspace.

#### 2.2.2. Spray Gun

**Figure 6:**
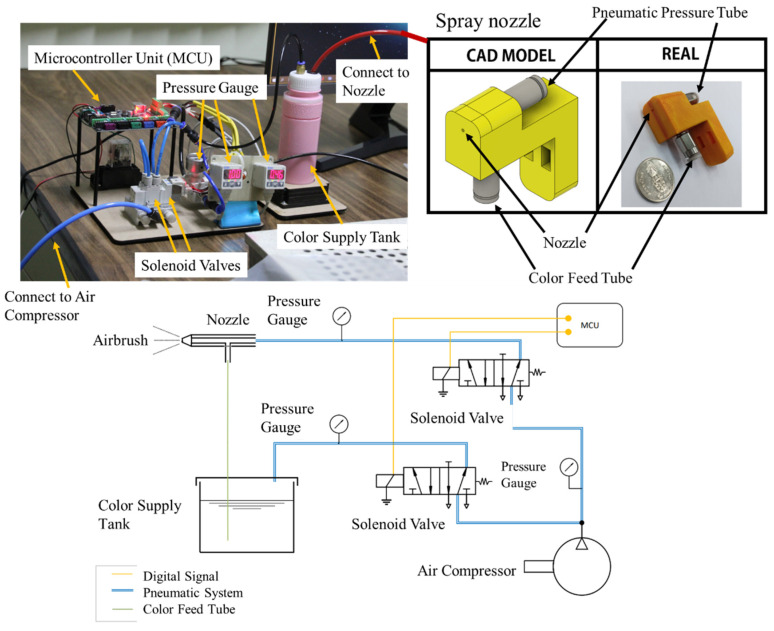
Spray Gun System and Architecture.

An automatic spray gun was used for painting and attached to the robot end-effector. The developed automatic spray gun for this project is shown in Figure 6. It comprises a spray nozzle, solenoid valves, an air compressor, pressure gauges, a color supply tank, and a microcontroller unit.

The spray nozzle was used as an airbrush and its nozzle diameter was 1 mm. The spray nozzle was connected to a color feed tube for supplying color from a color supply tank and to a pneumatic pressure tube with an operating pressure ranging from −0.95 to 10 bar. In addition, it had a 2-cfm consumption, 100-cc paint capacity, 40-mL/min fluid flow, and 100-mm spout distance. The volume of the color supply tank was 500 cc. Then, the solenoid valves—5/2 control valves—were used to control the pneumatic pressure connecting to the spray nozzle and color supply tank and were controlled by a microcontroller—MKS GEN L V1.0 Controller Board. The compressor for actuating the spray gun was a 0.34-KW air compressor. The air compressor had an 8-bar pressure, 32-L/min output volume, and 25-L tank. Then, the pressure gauges were used to monitor the pressure. The paint in the color supply tank can be any liquid, liquefiable, or mastic composition. After applying the paint in a thin layer, the paint was converted to an opaque solid film.

#### 2.2.3. Sensor Camera

An RGB camera—a Logitech C922 pro webcam [46]—was used as a sensor for the painting application. There were two purposes for using this camera. First, the camera was used to measure the target painting plane. The image processing technique was required to detect the target plane with and without a specific marker. With a specific marker, a simple image processing technique can be used to detect a plane. However, without the specific marker, it is difficult to detect the target plane and may be unsuitable for this work. Advanced image processing techniques may be required. For this project, the specific marker was attached to the target plane, and the image processing was run on the edge computer program to detect the target plane. The second purpose was to detect the painting’s quality. The painting quality was used as the feedback signal for the collaborative painting robot. Thus, the robot can paint the target as the painting design. Moreover, the camera also had its twin. The image from physical and digital twins can be used to analyze the accuracy and correct the digital twin model.

#### 2.2.4. Virtual System

CoppeliaSim is a robot simulation software that provides a set of developing robot tools. It is used as a 3D virtual representation and to simulate the real-time characteristics and motion of the real collaborative painting robot. Using this physics simulation, many advantages are obtained, such as forward and inverse kinematics, path planning, collision detection, and minimum distance calculation modules. Moreover, the collaborative robot model already exists. Therefore, these basic tools do not need to be developed. The CoppeliaSim version 3.0 rev3 edu was used for this development.

**Figure 7:**
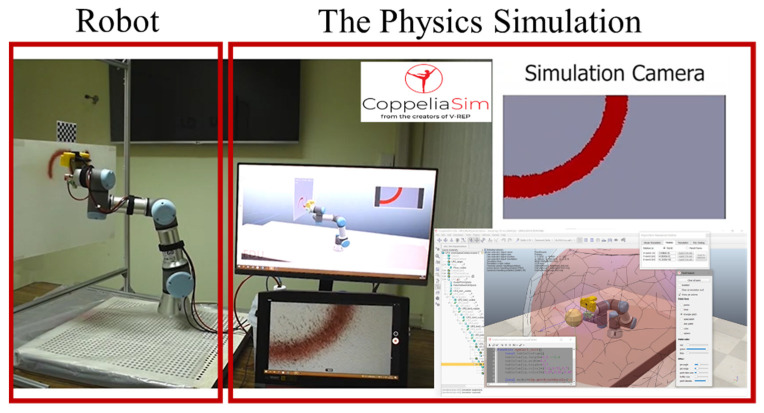
The Physics Simulation Program.

To implement the physics simulation as the digital twin, a script to run the simulation was required. The developed script received the robot input motion commands and data environment to run the simulation. Then, the simulation generated the robot outputs and sent them to the edge computer program to process the action. Moreover, the models of spray guns and sensor cameras were developed on the physics simulation. Thus, the physics simulation can simulate how the spray gun works and how the sensor camera views the environment and scene. These are useful for operators to visualize the outputs and for feedback signals of the painting system. The microcontroller shown in Figure 6 is an Androino board and is used to control the solenoid valves with input/output ports. It is also responsible for communication with a PC with a serial port. Figure 7 shows the physics simulation program working with a real robot.

#### 2.2.5. Control Modules

The real robot–virtual robot–user interface was developed with an edge computer script, which was developed on the MATLAB R2021a version. Operators can put the design painting and requirements in the script. Then, the script sends the requirements to simulate the situation in the physics simulation. After the simulation is confirmed, the physics simulation sends commands to the real robot. Moreover, the script receives the data from the sensor camera as the feedback signals to adaptively control the real robot and simulation. With this technique, the real-time simulation and control of the collaborative painting robot can be achieved. In addition, an online automated tool planning can be developed, and the painting quality can be controlled.

In this study, the painting process was controlled with interactive scripts. The primary script utilizes low-level commands from the 1. standard environment, 2. image processing, and 3. instrument control toolboxes. The block scripts were developed for the 2D spray path generator for a desired robotic configuration pattern and target plane pose; the target camera plane posed a language translator and communication with the collaborative robot controller and the physics simulation program. Then, the primary script that controls the block scripts was developed.

## 3. Experiments and Results

### 3.1. Painting Case Study and Experiments

With the growing construction requirements to fulfill the need for industrialization and housing, painting jobs are also growing. With multistory buildings, mega-structures, and restricted time for completing the project, the automated method of painting is the need of the hour. To evaluate and present the collaborative painting robot with the digital twin concept, a painting scenario was set up. In the scenario, an operator designs painting patterns, such as basic shapes and letters and their sizes. Next, the operator selected the painting location. Then, the collaborative painting robot generated automated tool planning and showed how the robot creates painting results. The operator can view simulated results and has an option to confirm or cancel a result. If the operator confirms a result, the real robot will run and complete the task. Notably, the confirm or cancel process of operators can be skipped if the operator does not require to confirm the result.

With the scenario, three painting patterns were used to evaluate the painting system. The first pattern is a square shape, the second one is an infinity symbol, and the third one is the word “CU.” The first pattern was used to evaluate the size of the painting pattern along the vertical and horizontal coordinates independently. The second pattern was used to evaluate the size of the painting pattern along the vertical and horizontal coordinates simultaneously. The third pattern was used to present the application of the collaborative painting robot at various painting locations. The text was designed and painted. Next, each pattern was performed five times. The results are shown in Section 3.2.

### 3.2. Results

**Figure 8:**
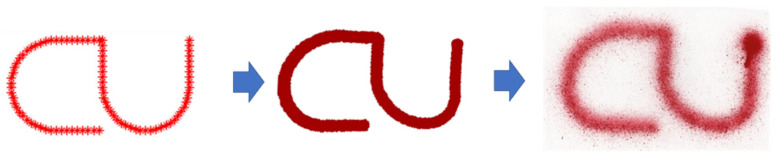
Designed Scenario of the Collaborative Painting Robot.

According to the designed scenario, the operator can design simple patterns and their sizes and implement them in the script. Then, the collaborative painting robot runs the simulation and the real robot. Figure 8 shows a designed scenario. Next, the sensor camera is used to determine the specific painting location, and the robot executes the task. The painting results are shown in the following section.

### 3.3. First and Second Pattern Results

**Figure 9:**
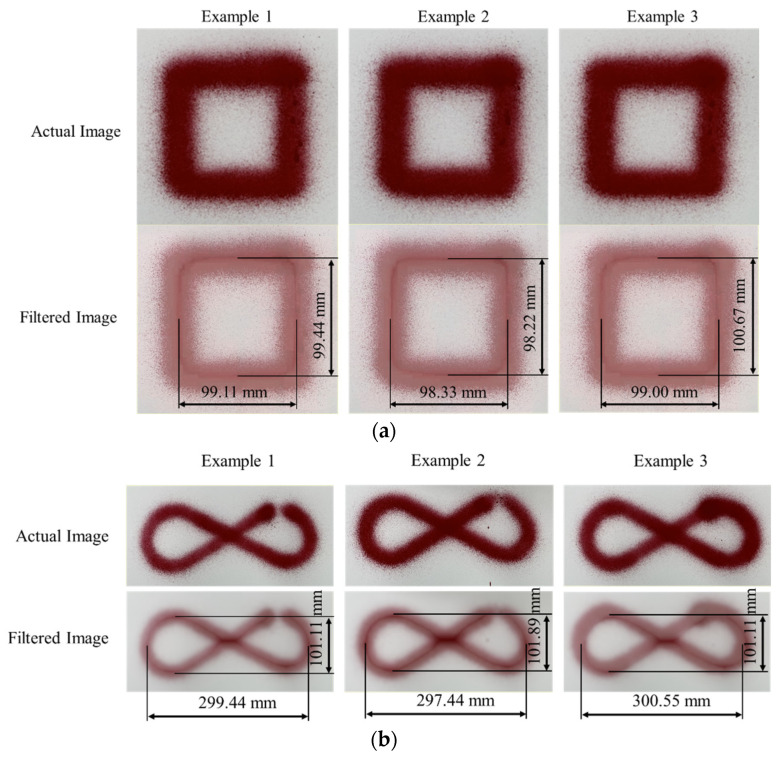
Examples of the Patterns: (**a**) Square Shape and (**b**) Infinity Symbol.

The square shape and infinity symbol patterns were painted five times, which were captured by a camera. The images were scanned using a scanner, and the shapes were analyzed with an image processing program—MVTec Halcon 20.11 [47]. Examples of the square shape and infinity symbol patterns are shown in Figure 9.

**Figure 10:**
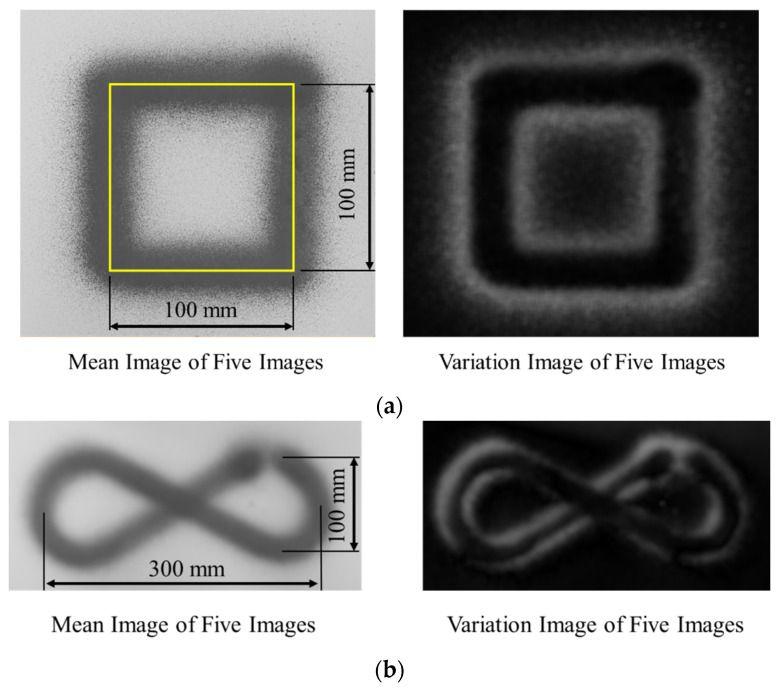
The Mean and Variation Images of Patterns: (**a**) Square Shape and (**b**) Infinity Symbol.

The sizes of the designed square shape and infinity symbol patterns are 100 × 100 and 300 × 100 mm^2^, respectively. The images of square shape and infinity symbol painting were used to create a variation model that can be used for image comparison by MVTec Halcon 20.11. The mean and various images of the images were generated and are shown in Figure 10. Because the mean images in Figure 10a,b have the same shape and size as the designed square shape and infinity symbol patterns, each painting line of square shape and infinity symbol is almost coincident. Moreover, the black in the variation image presents the area that has a small variation of the object position in the image, and the white inside it presents the area that has a large variation of the object position in the image. The variation image in Figure 10 shows that the black area forms the same shape as the designed square shape and infinity symbol patterns and the white area is around the square shape and infinity symbol patterns, meaning that the painting system has good repeatability. Further, the root mean square errors (RMSEs) of the square shape and infinity symbol patterns are listed in Table 1.

The RMSEs of the vertical and horizontal lines are computed based on the images; they are listed in Table 1. The standard deviations of the RMSEs of the vertical and horizontal coordinates are very small, confirming that the system has good repeatability. In addition, the mean RMSEs are small. Then, the color intensity measurement of square shape and infinity symbol patterns is shown in Table 2.

### 3.4. Third Pattern Results

**Figure 11:**
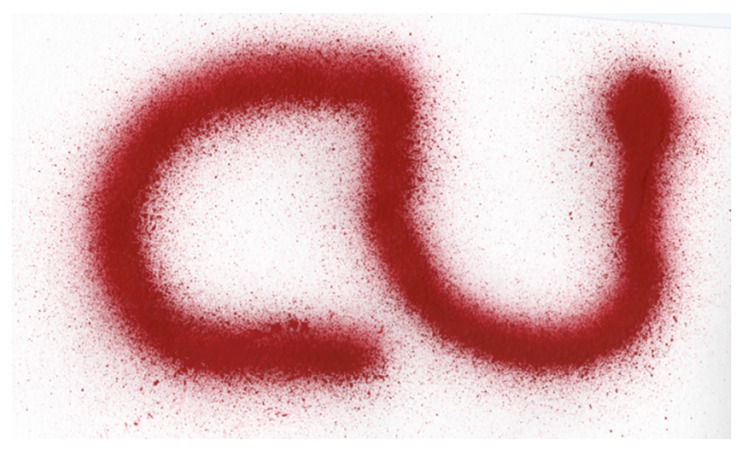
An Application Example.

The third pattern is an application of painting the word “CU” pattern (Figure 11). Then, the robot is allowed to paint the word in a variety of locations. These patterns show that the system can quickly create a simple painting text of shape.

## 4. Discussion and Future Recommendations

In this paper, we have demonstrated the concept of a digital twin for the case of a collaborative painting robot. The proposed digital twin architecture and communication model is an open architecture that supports available tools, hardware, and software to build a digital twin for a collaborative robotic cell. WiFi was used to communicate among all entities. The physics simulation was used to simulate the characteristics and motion of a real collaborative painting robot. The edge computer, the real robot and the camera were used to provide input to the physics simulation.

To evaluate the performance of the developed framework, a painting environment is setup. It is desired to see the performance of the collaborative painting robot at drawing basic shapes and letters in particular font sizes. As a test case, a square shape, an infinity symbol, and the word “CU” were given to the painting robot to draw/paint. According to the experiment and results, the collaborative painting robot with the digital twin concept can achieve these three basic tasks of painting. The operator can design simple patterns and let the robot generate an automated tool for planning and show how the robot creates a painting result. Then, Table 1 shows that the root mean square error (RMSE) of the painting is less than 1.5 mm, the standard deviation of the RMSE is less than 0.85 mm. and the maximum error is less than 2.6 mm. In addition, the color quality of the paint of the patterns is acceptable based on the visual inspection. Additionally, based on the color intensity measurement in Table 2, the color of the patterns is uniform and the color intensity can be controlled because the standard deviation of average color intensity is very low. Thus, the results demonstrate the success of our experiment.

As our proposed collaborative painting robot is in the development stage and there are indeed ways to improve its efficiency, there are certain limitations associated with it. The collaborative painting robot is currently set up and fixed on a table. Thus, the robot workspace is limited. It cannot paint outside the workspace. However, it is possible to put the system on a mobile robot to resolve this issue. With a mobile robot, the collaborative painting robot can move to different locations and paint. Currently, a sensor camera is used to measure the target painting plane. Because the camera is an RGB camera, it cannot detect the target plane well without any markers as there is no feature to detect to determine the target plane. Thus, a marker is required for the robot. However, it is possible to detect the target plane without a marker. The RGB camera needs to be replaced with a depth camera, such as an Intel Realsense D435i camera. The depth camera can provide a depth image to accurately determine the target plane.

Besides using the sensor camera to detect the target plane, it is used to detect painting quality. The painting quality is used as the feedback signal for the collaborative painting robot. Thus, the robot can paint the target as the painting design. Moreover, in the future, it would be interesting to create the digital twin level of the sensor camera. Currently, the camera is only a digital model level in the physics simulation. The camera in the physics simulation is just used to view what the robot sees. However, if the digital twin level can be implemented with the sensor camera, many features can be implemented, such as image processing and data/feature extraction, on the digital twin images. As such, various benefits are realized. The example of a vision-based data reader is shown in [14].

The developed spray gun system is simple and works well. However, the designed spray nozzle can still be improved for uniform liquid flow. A stereolithography 3D printer can be used as it has better accuracy. A porous nozzle can be easily created. Another solution is to use a standard commercial painting nozzle. Then, the flow of liquid can be easier to control. Currently, the spray gun is only a digital model level in the physics simulation; in addition, the spray gun model is a simple painting/line model and does not include the rheology of the paint. If the digital twin level can be implemented with the spray gun, many effects of paint can be simulated, such as diffusion and dispersion of paint. Then, the painting quality will be increased. Moreover, if the digital twin level of the robot, sensor camera, and spray gun are perfectly combined, a complicated painting task can be achieved because the painting system can accurately predict the painting quality.

The major benefits of implementing the digital twin framework are the improvement of costs, waste and time. This framework will be more efficient with low batch production. For example, advertising poster production with low batch production may produced less than 10 posters. In this case, the manual setup time may be more than 3 h. The operator with experience needs at least two sets of material for setting up the robot. Once the setup is finished and the process is running, if a painting error happened during the process without monitoring, all the products after the error happened are failures. Thus, the setup and operating cost, waste, and setting time of the traditional painting robot are high. However, with the digital twin framework, the setup time can be reduced to less than 1 h because the operators can quickly simulate the results without waiting for the robot to paint and they can evaluate many scenarios for the painting setup. Then, only one set of materials for setting up the robot may be enough because the simulation is used to confirm the process. Additionally, during the production process, if a painting error happened, the process can be stopped or corrected to prevent product failure. Therefore, the digital twin framework can reduce the the setup and operating cost, waste, and setting time in this case.

## 5. Conclusions

A collaborative painting robot was developed using the digital twin framework. The prime advantages of the development of this product are improving the safety and health of workers, reducing waste material and effectively getting the job done. The prototype spray mechanism, with its digital model, was developed for the robot’s end effector as part of the project. Digital versions of the robot and an eye-in-hand camera are commercially available. The physics simulation is loaded with all the digital models in the proposed painting robotic cell and is thus used as a digital shadow. The proposed digital twin architecture and communication are effective and flexible. In this study, the edge computer is used to control the process flow, communicate with the robot’s controller, perform virtual simulation and data storage, process images from the physical camera, and generate a 2D path for the robot. This concept makes extensive use of enabling technologies and tools to build a painting robot and its digital twin. As a result, painting can be practiced in a virtual environment where the simulated process and outcome are observed before the real execution. The results of the letter and basic shape experiments demonstrate the effectiveness of the proposed techniques. The proposed architecture is an open architecture that allows for future exploration to provide additional benefits, such as: (1) finding a solution when the fault begins; (2) refining the control or optimizing operation; and (3) using historical data for planning. For future work and improvement in collaborative robot performance, the adaptive control concept can be introduced into the system. Further enhancement can be made if the live image is compared to the digital twin image and the estimated error can be fed to the system for correction. Machine learning can also be used to train the collaborative painting robot about the expected error so that the painting can be further enhanced.

## Figures and Tables

**Table 1:** Root mean square error (RMSE) of square shape and infinity symbol patterns.

RMSE (Pixels)	Square Shape		Infinity Symbol	
	Vertical	Horizontal	Vertical	Horizontal
Mean of RMSE (mm)	1.01	1.15	1.50	1.49
Standard Deviation of RMSE (mm)	0.63	0.35	0.67	0.84
Maximum RMSE (mm)	1.78	1.67	2.22	2.56

Note: 1 mm is approximately 9 pixels, or 1 pixel is 0.1111 mm.

**Table 2:** Color intensity measurement of square shape and infinity symbol patterns.

	Square Shape			Infinity Symbol		
	Red Channel	Green Channel	Blue Channel	Red Channel	Green Channel	Blue Channel
Average Color Intensity	85.78	5.82	2.26	85.35	5.67	1.32
Standard Deviation of Average Color Intensity	3.83	3.58	3.22	3.23	3.19	1.25

Note: The color intensity is measured based on 8 bits images.

## Data Availability

Not applicable.
